# Scanning-Based Dynamic Mask Projection for Ultrafast Laser Ablation of Thin Films

**DOI:** 10.3390/nano16040262

**Published:** 2026-02-17

**Authors:** Jonas Amann, Markus Kircher, Andreas Otto, Balint Istvan Hajas, Alexander Kirnbauer, Justas Baltrukonis, Roland Fürbacher

**Affiliations:** 1 Institute of Production Engineering and Photonic Technologies, TU Wien, Getreidemarkt 9, 1060 Vienna, Austria; markus.kircher@tuwien.ac.at (M.K.); roland.fuerbacher@tuwien.ac.at (R.F.); 2 Institute of Materials Science and Technology, TU Wien, Getreidemarkt 9, 1060 Vienna, Austria; balint.hajas@tuwien.ac.at (B.I.H.); alexander.kirnbauer@tuwien.ac.at (A.K.); 3 Ceramic Data Solutions GmbH, Salzfertigergasse 3, 4810 Gmunden, Austria

**Keywords:** laser ablation, femtosecond laser, digital micromirror device, mask scanning, mask projection, thin film, tantalum nitride

## Abstract

Ultrafast laser processing is constrained by an inherent throughput–resolution trade-off, typically addressed either by high-speed single-beam scanning or by parallel processing approaches. Here, a scanning-based dynamic mask projection concept is presented, combining both strategies by integrating a digital micromirror device (DMD) for dynamic binary amplitude mask generation with galvanometric scanning for high-speed lateral repositioning of the projected pattern. A high-numerical-aperture microscope objective is used to project the mask for thin film laser ablation with sub-micrometer feature sizes, while scanning extends the processing area beyond a single projected pattern, ultimately limited by the objective’s field of view. The concept is demonstrated by selective single-pulse pattern ablation of 10 nm thick tantalum nitride (TaN) thin films on glass substrates using 230 fs pulses at a center wavelength of 515 nm. The optical system enables a 770 nm minimum feature size across a scan field with an area-equivalent circular diameter of 550 µm. Dynamic mask projection combined with fast scanning offers a scalable route to high-throughput laser nanoprocessing and is relevant to fabrication and processing of nanomaterials, digital mask lithography, and micro- and nanomachining.

## 1. Introduction

Ultrafast laser processing has matured into a versatile micro- and nanofabrication tool [1,2], achieving subdiffraction-limited spatial resolution [3,4] with minimal heat-affected zones [5,6]. One of the key challenges of this technology’s application remains the trade-off between spatial resolution and fabrication throughput [1]. Conventional single-beam processing attains nanoscale features but scales poorly to large areas because structures are generated serially. To address throughput and efficiency, prior work has predominantly relied on high-speed beam steering techniques such as galvanometer scanners [7,8], polygon scanners [9], acousto-optic deflectors [10,11], or their combination [12], offering speed advantages over translation stages. Beyond scanning and beam-steering speeds, throughput in serial laser processing is fundamentally limited by the repetition rate of the laser source. Parallelization approaches such as static diffractive beam-splitting to generate arrays of spatially separated beams [13,14] and Direct Laser Interference Patterning (DLIP) [15,16] help overcome this limitation and are widely used to enhance throughput, although they are usually restricted to specific static patterns. More recently, dynamic beam shaping has emerged as a powerful route, providing rapid, on-the-fly reconfiguration of the projected intensity distribution without mechanical mask exchange. Spatial Light Modulators (SLMs) allow for projecting arbitrary intensity patterns, enabling additive manufacturing [17,18,19], selective ablation [20,21], laser-induced forward transfer applications [22], and laser lithography [23,24,25,26,27,28]. In high-repetition-rate ultrafast laser processing using dynamic beam shaping, the achievable throughput can become limited by the modulator switching frequency. Liquid-crystal phase modulators are typically limited to switching frequencies on the order of 120 Hz, restricting the pattern update rate accordingly. Consequently, pulse-synchronous operation is not feasible at kHz–MHz laser repetition rates. In contrast, dynamic binary amplitude modulators (micro-opto-electromechanical systems), such as Texas Instruments Digital Micromirror Devices (DMDs), provide switching frequencies in the kHz regime, enabling synchronization with the laser repetition rate for high-speed nanoprocessing.

This study introduces a scanning-based dynamic mask projection system that combines a DMD-defined binary amplitude mask with high-speed galvanometric scanning to enhance the processing area and throughput. The mask pattern is demagnified and imaged onto the sample through a high-numerical-aperture objective, while the galvanometer scanner rapidly repositions the projected pattern within the objective’s field of view. Single-pulse exposures at these discrete positions enable selective ablation of the arbitrary patterns. The resulting hybrid architecture inherits the strengths of both components: the dynamic mask provides rapid, parallel multi-spot pattern shaping, while the galvanometer scanner extends the processing area and allows for high-speed pattern positioning.

Using 10 nm tantalum nitride (TaN) films on glass as a model material system for thin film ablation, rapid and precise selective ablation with sub-micrometer resolution is demonstrated, and the minimum achievable feature sizes and the scanning field (processing area) are quantified. In addition, an analysis of the optical constraints and the prospective scalability of the design is conducted by evaluating the dependence of the system’s performance on the digital dynamic mask parameters and projection optics specifications (numerical aperture, field of view, and aberration correction) as well as material response, including ablation-threshold behavior. Fourier-optical modeling, coupled with theoretical approximations, is used to predict feature sizes and processing windows. To parameterize the threshold-limited ablation regime, the single-pulse ablation threshold of 10 nm TaN on glass is determined using 230 fs pulses at 515 nm.

## 2. Materials and Methods

### 2.1. Working Principle of Scanning-Based Dynamic Mask Projection

**Figure 1:**
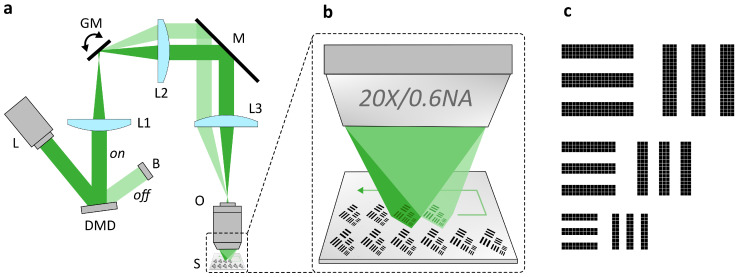
(**a**) Schematics of experimental setup including: fs-laser (L), Digital Micromirror Device (DMD), beam dump (B), first relay lens (L1), galvanometer mirrors (GM), second relay lens (L2), folding mirror (M), tube lens (L3), objective (O), sample (S). (**b**) Schematic illustration of the scanning-based dynamic mask projection procedure. The projected mask pattern is positioned on the thin film sample through the deflection of the galvanometer scanner and ablated by single fs-laser pulse irradiation. (**c**) Schematic line pattern comprising line structures with both horizontal and vertical orientations corresponding to the DMD mask or micromirror configuration, consisting of lines sized 2×10, 3×15, and 4×20 pixels whereby a black pixel corresponds to a micromirror in “On” state.

Femtosecond laser pulses are spatially amplitude-modulated using a DMD-based digital dynamic mask. As in conventional optical projection systems, commonly employed in laser lithography or laser additive manufacturing, the pattern is projected by an infinity-corrected microscope imaging system. The configuration presented here extends this concept by incorporating two additional lenses after the DMD, which relay the pattern to an intermediate image plane, with a galvanometer scanner positioned near the Fourier plane between the two lenses, as illustrated in Figure 1. The intermediate image is then demagnified and projected onto the sample by a tube lens and a high-NA objective. The galvanometer scanner enables scanning of the projected pattern across the sample. By synchronizing the deflection of the two galvanometer mirrors with the laser pulse emission and DMD pattern switching, the mask pattern can be projected and precisely positioned on the sample. Pulse-to-pattern synchronization is implemented by using the scanner/motion controller as timing master. At predefined scan coordinates, the controller outputs a position-synchronized TTL signal that is routed to the laser trigger input for pulse-on-demand emission and can be provided simultaneously to the DMD controller as an external trigger to advance a pattern sequence. Single-pulse irradiation at each scan position enables selective ablation of the pattern. Due to the high switching frequency of the DMD, the high repetition rate of the laser and the fast deflection speed of the galvanometer scanner, this process can be performed at high speed, facilitating rapid and large-area processing of thin films.

### 2.2. Optical Setup and Design

The femtosecond laser source (Carbide-CB5 with harmonics module, Light Conversion, Vilnius, Lithuania) used in this study was operated at a central wavelength of 515 nm, with a pulse duration of 230 fs and a maximum pulse energy of up to 42 µJ. The Gaussian output beam was characterized by a diameter of 2.4 mm and a beam quality factor of M^2^ < 1.07. The digital dynamic mask (DLP7000 DMD, Texas Instruments, Dallas, TX, USA) features a pixel pitch of 13.68 µm, a resolution of 1024×768 pixels, a fill factor of 92% and a ±12° micromirror tilt angle. The square micromirrors are arranged in a diagonal geometric grid. The angle of incidence was experimentally optimized to maximize the DMD’s single-order diffraction efficiency and thereby the effective utilization of the laser pulse energy [29]. In the final configuration, the device was illuminated at an incidence angle of 36° with respect to the surface normal. For a uniform DMD pattern with all micromirrors in the “On” state (−12° micromirror tilt), a diffraction efficiency of 62% into the selected diffraction order was measured. Under these conditions, the beam reflected from micromirrors in the “On” state propagates at 12° with respect to the device normal and is aligned with the optical axis of the subsequent projection system, while light from micromirrors in the “Off” state is directed towards larger angles and can be blocked by a beam dump. Two 2″ plano-convex lenses (f = 200 mm) were used to generate an intermediate image of the DMD pattern in a 4f optical imaging system configuration, whereby the galvanometer scanner (AGV10HPO-BE1-W11-TAS, Aerotech, Pittsburgh, PA, USA), positioned close to the Fourier plane between them, introduced angular beam deflection, resulting in a lateral displacement of the image. To prevent damage from high fluences, the beam was focused by the first lens to a position 40 mm before the first mirror of the galvanometer scanner. This axial offset resulted in a minor lateral displacement of the beam at the entrance pupil of the objective. The intermediate image of the DMD pattern was projected onto the sample plane using a high-NA microscope objective (20X EO HR Infinity Corrected Objective, 0.60 NA, Edmund Optics, Barrington, NJ, USA) in combination with a 2″ plano-convex tube lens (f = 300 mm). The tube lens and objective were separated by 320 mm, slightly exceeding the nominal tube lens focal length, to reduce the fluence at the objective and thereby mitigate the risk of laser-induced damage. This configuration yielded a total system demagnification of M=1/30 and an effective scanning speed at the sample plane of approximately 200 mm/s.

A resolution test pattern, shown in Figure 1c, similar to the USAF 1951 resolution target, was employed for the ablation experiments. The pattern comprises horizontal and vertical line features with multiple linewidths. Across the test pattern area, covering 800×650 µm^2^ on the DMD, the laser profile intensity variation remained below 30%. On the DMD, the pattern included linewidths of 27.36 µm, 41.04 µm, and 54.72 µm, implemented using line segments composed of 2×10, 3×15, and 4×20 micromirrors, respectively. The projected structures on the sample surface, estimated by purely geometrical demagnification, exhibited linewidths of 0.91 µm, 1.37 µm, and 1.82 µm.

For compactness and optical path routing, the setup included six folding mirrors. For clarity, only one folding mirror is shown in the simplified schematic Figure 1a. The total optical efficiency of the system was 45%, defined as the ratio of the optical power measured at the sample plane to that at the laser output, determined using a uniform DMD pattern with all micromirrors in the “On” state. The dominant loss contributions arise from the DMD’s limited diffraction efficiency and the finite reflectivity of the mirrors.

### 2.3. Fabrication of TaN Thin Films

The coatings were synthesized by non-reactive DC magnetron sputtering of a powder-metallurgically produced stoichiometric TaN compound target (Plansee Composite Materials GmbH, Lechbruck am See, Germany) in a modified Leybold Heraeus Z400 PVD machine in a bottom-up configuration. The 200 µm thick glass substrates (Gorilla Glass, Corning Incorporated, Corning, NY, USA) were aligned parallel to the target above the racetrack at an affixed distance of 40 mm. After evacuating the sputter chamber down to a base pressure below $1\times 10^{-4}$ Pa, the samples and chamber were degassed by heating the substrate holder up to 400 °C for at least 20 min. Before mounting these samples to the substrate holder within the machine, they were cleaned in an ultrasonic bath of acetone and isopropyl alcohol for 5 min, respectively. After the required substrate temperature was reached, the chamber was purged with Ar, and the samples were sputter cleaned with −150 V pulsed bias at an Ar pressure of 2 Pa to remove residuals and contaminants from their surfaces and to energetically activate these [30]. Sputter-cleaning of the target was conducted behind a closed shutter for additional 5 min at the chosen parameters to prepare the coatings, after which the shutter between the substrate holder and the target was removed to start the deposition. The samples were prepared in constant DC mode with a current of I=0.3 A and a power of P=110 W, while a constant bias potential of $U_{b}=-50$ V was applied to the substrate holder.

### 2.4. Ablation Characterization

The ablated features were examined using optical microscopy (S Neox, Sensofar, Terrassa, Spain), taking advantage of the pronounced intensity contrast between the reflective TaN thin film and the exposed glass substrate. Images were acquired using high-NA objectives (M Plan Apo NIR 10X, 0.26 NA and M Plan Apo NIR HR 100X, 0.70 NA, Mitutoyo, Kawasaki-shi, Kanagawa, Japan) with a minimum lateral resolution of approximately 400 nm. High-resolution imaging was performed by field-emission scanning electron microscopy (Quanta 250 FEG, FEI (Thermo Fisher Scientific), Hillsboro, OR, USA). Samples were mounted on aluminum stubs; the TaN film was electrically conducted to the stub using silver paint along the sample edge. Imaging at a working distance of 7.3 mm and a beam current of 5 kV was used to resolve nanometer features, edge morphology, and debris/redeposition.

### 2.5. Scan Field Characterization

The scan field denotes the processing area in the workpiece plane over which a steered beam or projected, scanned pattern can be focused and ablated while satisfying a defined minimum resolution or feature size. The usable scan field was quantified by identifying the area using optical microscopy within which the projected pattern was reliably ablated at the specified resolution. To quantify the relative positioning precision between two adjacent projected patterns (pattern stitching), ablated line-pattern structures within a 500×500 µm^2^ area were analyzed, and the coordinates of one reference point per projected pattern were extracted from microscopy images. The measured reference-point position on the pattern grid is denoted by the 2D vector $r\left(m,n\right)={\left[x(m,n),y(m,n)\right]}^{T}$, where *m* and *n* are the integer column and row coordinates of the pattern grid. To separate deterministic field distortion from local positioning variability, a smooth second-order polynomial mapping was fitted to the measured coordinates, yielding model-predicted positions $\hat{r}\left(m,n\right)$. The local stitching-error was then computed as the residual nearest-neighbor displacement after subtracting the fitted field geometry, for neighbors along the scan-line direction (m,n)→(m+1,n) and between adjacent scan lines (m,n)→(m,n+1). This separates neighbor pairs along the scan-line direction from pairs orthogonal to it, which can be affected differently by scanner dynamics and cross-axis coupling. The scan-direction stitching-error vector is defined as:

(1) $$\delta d^{\left(\mathrm{scan}\right)}\left(m,n\right)=\left(r(m+1,n)-r(m,n)\right)-\left(\hat{r}\left(m+1,n\right)-\hat{r}\left(m,n\right)\right),$$

and the line-to-line stitching-error vector as:

(2) $$\delta d^{\left(\mathrm{line}\right)}\left(m,n\right)=\left(r(m,n+1)-r(m,n)\right)-\left(\hat{r}\left(m,n+1\right)-\hat{r}\left(m,n\right)\right).$$

For each neighbor class, the stitching resolution is reported as the 1σ standard deviation of the stitching-error components. Field dependence was assessed by partitioning the (m,n) grid into three nested index regions: (i) a central region defined by the middle 50% of the index span in both *m* and *n*, (ii) an intermediate region defined by the middle 75% excluding the central region, and (iii) an outer region comprising the remaining indices. Stitching-error statistics were computed from neighbor pairs fully contained in each zone (>40 pairs per zone).

### 2.6. Femtosecond Ablation Threshold Characterization Method

For selective femtosecond laser ablation, 10 nm TaN thin films on glass substrates were used as a representative material system. To parameterize the threshold-limited ablation regime and to define the experimental processing window, the single-pulse ablation threshold fluence was determined. Characterization was performed using 230 fs pulses at a center wavelength of 515 nm, following Liu’s [31] linear regression method. The laser pulses were precisely focused onto the sample surface using a microscope objective (M Plan Apo NIR 10X, Mitutoyo, Kawasaki-shi, Kanagawa, Japan). The laser beam pulse energy was controlled by an external attenuator and the laser power was measured after the focusing lens using a photodiode power sensor (PD-50-D9-UV, Laserpoint Srl, Vimodrone, Italy). Pulse energy was calculated by dividing the average laser power by the corresponding repetition rate. A linear regression was performed and the threshold fluence was determined by the dependence of the squared diameter of the spots on the peak fluence according to:

(3) $$D^{2}=2\omega_{0}^{2}\cdot ln\left(\frac{F}{F_{th}}\right),$$

where *D* is the spot diameter, $\omega_{0}$ is the beam waist, *F* is the peak fluence, and $F_{th}$ is the threshold fluence. The peak fluence was obtained from F=E/EBA, where *E* denotes the pulse energy and the effective beam area is given by $\mathrm{EBA}=\pi \omega_{0}^{2}/2$ [32]. This widely used technique simplifies testing and enables the determination of the beam waist (≈5 µm). The sample’s surface was irradiated with single pulses at 20 sites across the sample and the pulse energies were varied gradually across discrete levels. For the optical microscopy images acquired after laser irradiation, circles were fitted to the damaged regions using image analysis software (SensoVIEW (v1.9.1), Sensofar, Terrassa, Spain) and the resulting diameters were employed as the response variables in the regression model above. Uncertainties in the ablation threshold measurement were calculated using Gaussian error propagation, incorporating standard errors and covariance from the linear regression based on a least squares approach, as detailed by Taylor [33].

### 2.7. Fourier-Optical Simulation of DMD Pattern Projection

Fourier-optical modeling provides predictions of the resolution limits of projected patterns and enables comparison of experimentally measured feature sizes with simulation results. A binary amplitude mask representing the “On” and “Off” states of the DMD’s micromirrors is implemented (micromirror pitch 13.68 µm; array fill factor 92%). Under coherent illumination, the simulated mask pattern is relayed by a 4f system modeled with ideal lenses of focal lengths $f_{1}=300$ mm and $f_{2}=10$ mm, yielding a magnification factor of $M=f_{2}/f_{1}=1/30$.

Accordingly, the mask pattern is Fourier transformed and an ideal circular pupil with a diameter of D=12 mm, corresponding to the objective’s aperture stop diameter, is applied in the Fourier plane of the first lens. Physical coordinates ($x_{\nu }$, $y_{\nu }$) in this plane relate to spatial frequencies via $\nu_{x}=x_{\nu }/\left(\lambda f_{1}\right)$, $\nu_{y}=y_{\nu }/\left(\lambda f_{1}\right)$, where λ denotes the wavelength [34]. The demagnified image is then obtained by an inverse Fourier transform. For the numerical implementation, the complex input field is sampled on a 2048×2048 grid over a window (2×2 mm^2^) that fully contains the active mask area (0.80×0.65 mm^2^) with a lateral sampling of Δx=Δy≈1 µm, chosen to avoid aliasing under the imposed pupil (fulfilling the sampling theorem $\Delta x\leq 1/(2\nu_{c})$ with cutoff-frequency $\nu_{c}=D/\left(2\lambda f_{1}\right)$ [34]). Computations were performed in Python (v3.12.0), Fourier propagation and masking were implemented with NumPy’s FFT routines, and visualization was conducted using Matplotlib (v3.10.7). Assumptions include scalar, coherent propagation in the paraxial regime and incorporates the DMD fill factor by representing each pixel with a reduced reflective micromirror area.

## 3. Results

### 3.1. Characterization of the TaN Thin Films

**Figure 2:**
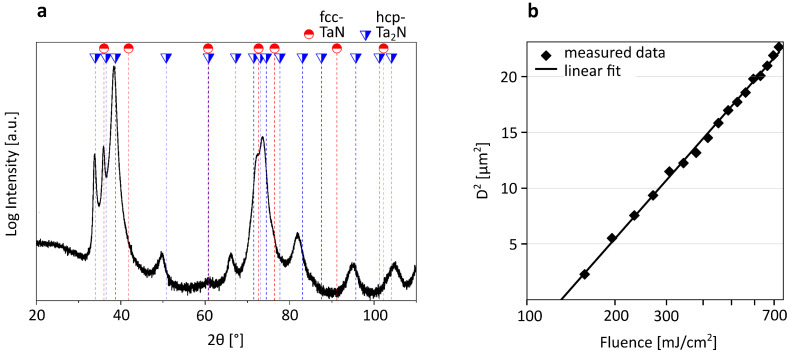
(**a**) XRD pattern of the 10 nm TaN thin film showing a large number of broad reflexes indicative of a fine-grained structure. Reference positions of diffraction planes for fcc-TaN (ICDD: 04-019-2403) and hcp-Ta_2_N (ICDD: 00-029-1321) are used to assist the XRD pattern evaluation. (**b**) Single-shot ablation threshold determination by Liu’s method for 230 fs, 515 nm pulses: measured squared spot diameter data (points) and linear fit (line) yielding a threshold of $F_{th}=(131.0\pm 0.8$) mJ/cm^2^.

XRD analyses were performed on 10 nm TaN thin film coatings on glass substrates with a material diffractometer (Empyrean, Malvern Panalytical, Almelo, The Netherlands) equipped with a Cu-K_α_ (λ=1.54 Å) radiation source. Reference positions of diffraction planes for fcc-TaN (ICDD: 04-019-2403) and hcp-Ta_2_N (ICDD: 00-029-1321) were used to assist the XRD pattern evaluation. XRD pattern of the coating is shown in Figure 2a. The diffractogram displays a large number of broad reflexes, a clear sign of fine-grain structure. The coating exhibits primarily an understoichiometric hcp-Ta_2_N structure, which can be expected with non-reactive deposition processes [35]. As there is no surplus of N_2_ during the sputter process, as could be the case in reactive processes, it becomes difficult to stabilize the stoichiometric fcc-TaN phase.

The single-shot ablation threshold of the samples was quantitatively assessed using Liu’s method [31], yielding (131.0±0.8) mJ/cm^2^ for 230 fs pulses with a central wavelength of 515 nm. The measured data points and the linear fit are illustrated in Figure 2b. Optical microscopy images of the ablated spots, along with the corresponding diameters and pulse energies, are provided in Appendix A.1. Other transition metal nitride thin films (TiN, CrN) exhibit an ablation threshold in a similar range [36,37].

### 3.2. Thin Film Ablation by Scanning-Based Dynamic Mask Projection

**Figure 3:**
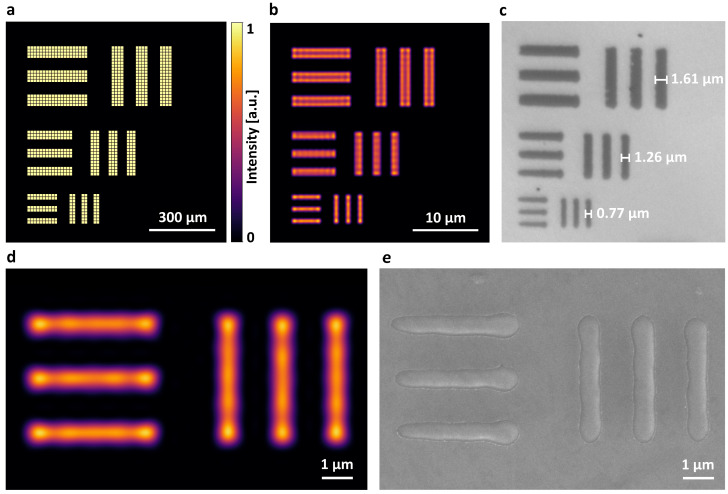
Simulated pattern projection and corresponding ablation results. (**a**) Binary amplitude mask at the DMD. (**b**) Simulated intensity of the demagnified pattern, accounting for the objective’s finite NA. (**c**) Optical microscopy image of the ablated pattern on a 10 nm TaN film; annotated linewidths are (0.77±0.05), (1.26±0.08), and (1.61±0.17) µm. (**d**) Detail of the simulation result for the 0.77 µm line pattern, illustrating the non-uniform intra-line intensity caused by the limited spatial resolution of the projection system. (**e**) SEM image of the ablated 0.77 µm line pattern corresponding to the simulation in (**d**).

Line patterns on 10 nm TaN thin film coatings on glass substrates were ablated to generate high-contrast sub-micron surface features at high positioning speed and accuracy. The expected feature sizes or linewidths *L* fabricated by the scanning-based dynamic mask projection process can be approximated by the demagnification of the projected pattern and a process-dependent parameter *k*, using the following expression [27]:

(4) L=N·l·M·k

where *N* is the number of pixels of the linewidth, *l* denotes the DMD’s micromirror pitch and *M* is the magnification factor. The parameter *k* (from 0 to 1) represents the ratio of the measured ablated line width to the projected line width predicted by purely geometrical demagnification and depends on the sample’s ablation threshold, the incident fluence, and the projected intensity distribution, including diffractive effects. The experimental results showed ablated linewidths *L* of (0.77±0.05), (1.26±0.08), and (1.61±0.17) µm, corresponding to k-values of (0.85±0.06), (0.93±0.06), and (0.89±0.09), respectively. The standard deviations were determined from statistical evaluation based on 40 individual measurements per linewidth with an optical microscope. The simulated pattern projection, accounting for the objective’s finite numerical aperture and diffraction, and the corresponding ablated structures on 10 nm TaN are shown in Figure 3. At the demonstrated limited resolution, the projected line patterns did not exhibit a uniform intra-line intensity distribution and showed more rounded and smoother features compared to the DMD’s pattern. The DMD pixel grid, set by the finite micromirror pitch and the non-unity fill factor, was not resolved in the sample plane. The ablated structures were consistent with the simulated field, with features confined to regions exceeding the ablation threshold.

The fluence incident on the dynamic mask $F_{mask}$ was estimated by dividing the measured pulse energy $E_{pulse}$ = 15 µJ by the effective beam area $\mathrm{EBA}=\pi \omega_{0}^{2}/2$, with a beam waist of $\omega_{0}=1.2$ mm. The corresponding fluence on the sample was obtained by scaling with the system magnification M=1/30 and accounting for the overall optical efficiency $\eta_{opt}=45$%. Based on these parameters, the resulting fluence on the workpiece was calculated as:

(5) $$F=\frac{F_{mask}}{M^{2}}\cdot \eta_{opt}=\frac{E_{pulse}}{\mathrm{EBA}\cdot M^{2}}\cdot \eta_{opt}\approx 270\;{\mathrm{mJ}/\mathrm{cm}}^{2}.$$

This value was approximately twice the material ablation threshold ($F_{th}$ = 131 mJ/cm^2^) and enabled reliable pattern ablation of the thin film. Higher pulse energies (up to 42 µJ in the present laser system) could be exploited by increasing the illuminated DMD area (larger EBA), enabling proportionally larger projected masks at comparable fluence.

For single pulse exposure, projected rectangular resolution test patterns with a size of approximately $A_{proj}=27\times 22$ µm^2^, rotated by 45° with respect to the x-y scan axes, were ablated with the chosen effective pattern period $D=27\cdot \sqrt{2}$ µm. The pattern rate *f* was approximated from the effective galvanometric scanning speed *v* = 200 mm/s and the spatial pattern period as f=v/D=5.2 kHz. The corresponding areal throughput *T* was estimated by the product of the pattern rate and the projected mask area:

(6) $$T=f\cdot A_{proj}=3.1\;{\mathrm{mm}}^{2}/s.$$

Under these conditions, the throughput exceeded that of single-spot scanning by at least a factor of 20 (considering single-spot scanning parameters of 0.77 µm spot diameter and 200 mm/s scanning speed).

### 3.3. Scan Field

**Figure 4:**
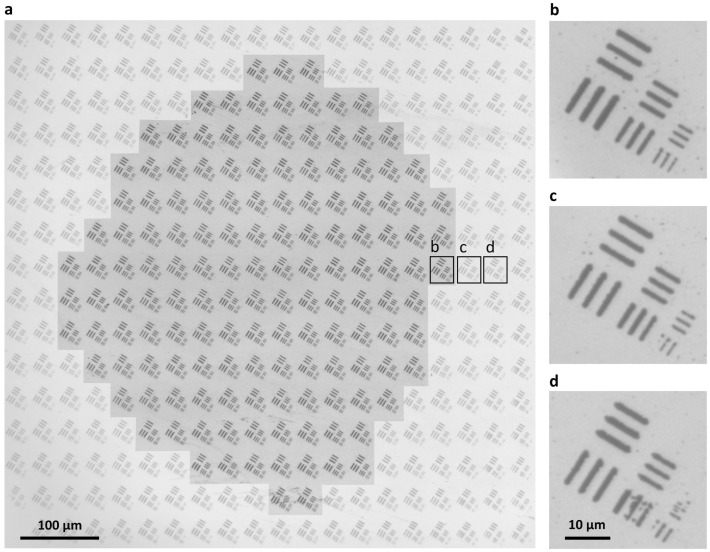
(**a**) A projected line pattern with linewidths of (0.77±0.05), (1.26±0.08), and (1.61±0.17) µm is scanned over a 10 nm TaN film and ablated with single-pulse irradiation. The central, highlighted area indicates the scan field, where linewidths down to 0.77 µm are resolved; the outer area indicates loss of resolvability due to field-dependent aberrations. (**b**–**d**) Ablated line patterns at different positions on the sample: within the scan field, all line widths are resolved (**b**), whereas outside the scan field the 0.77 µm (**c**) and 1.26 µm (**d**) features are no longer fully resolved. Resolution degrades progressively with increasing distance from the field center.

For dynamic mask projection, the scan field of the optics must exceed the projected pattern size to enable lateral scanning and thereby expand the processing area. Consequently, the attainable scan field is highly dependent on the numerical aperture of the projection optics, the objective’s designed field of view and system apertures along the beam path. Due to the typical corrections of microscope objectives and the additional spherical aberrations of the optical system’s lenses, diffraction-limited performance can only be expected on-axis. Off-axis, field-dependent aberrations and field curvature increase the spot size and reduce the resolvable linewidth. To assess the scan field, the projected pattern was scanned across the sample and ablated at successive positions with a pattern period $D=27\cdot \sqrt{2}$ µm within a region of approximately 980 µm effective diameter, here defined as the area-equivalent circular diameter due to the observed ellipticity (the full area is discussed in Appendix A.2). This upper bound was set by the clear apertures of the system’s mirrors and lenses as well as the objective’s field stop. For each of the pattern’s three target linewidths, the usable scan field was quantified by identifying the area within which the corresponding structures can be reliably ablated at the specified resolution. Area-equivalent scan field diameters of 550 µm, 740 µm, and 800 µm were obtained for minimum feature sizes of 0.77 µm, 1.26 µm, and 1.61 µm, respectively. The achievable resolution degraded toward the periphery of the scan field, consistent with increasing off-axis aberrations, as shown in Figure 4. Ellipticity of the scan field was observed and can be partly attributed to finite mirror spacing in the galvanometer scanner (resulting in non-identical scan axes) and to a focal-plane shift before the scanning mirrors. In addition to the field-dependent feature resolution, adjacent-pattern stitching resolution within a 500×500 µm^2^ area was quantified using nearest-neighbor stitching-error vectors after removing deterministic field distortion via smooth polynomial mapping of the measured pattern positions. Field dependence was evaluated by grouping neighbor pairs according to their location in the scan field into a central region, an intermediate zone, and an outer region, corresponding approximately to the inner 50%, 50–75%, and outer 25% of the evaluated pattern grid extent, respectively. In the central zone, the 1σ standard deviations of the stitching-error components in scan-line direction were $\sigma \left(\delta d_{x}^{\left(\mathrm{scan}\right)}\right)=0.190$ µm and $\sigma \left(\delta d_{y}^{\left(\mathrm{scan}\right)}\right)=0.188$ µm. For line-to-line neighbors (orthogonal to the scan-line direction), the standard deviations of the stitching-error components yielded $\sigma \left(\delta d_{x}^{\left(\mathrm{line}\right)}\right)=0.198$ µm and $\sigma \left(\delta d_{y}^{\left(\mathrm{line}\right)}\right)=0.177$ µm. Across all zones, the corresponding values stayed within 0.18–0.24 µm and did not show a monotonic increase toward the outer region.

## 4. Discussion

### 4.1. Feature Size and Resolution Limits

This section addresses the ablated features demonstrated in this work and estimates the minimum feature size attainable with the present dynamic mask projection system. Beyond pure geometrical demagnification, the analysis explicitly considers diffraction and the optical resolution limit.

The smallest structures achieved are ablated linewidths of 770 nm obtained from projecting a DMD line pattern with a linewidth of 27.36 µm (two micromirrors) at a demagnification of M=1/30. The finite pupil of the 4f relay imposes circular apodization and low-pass spatial filtering in the Fourier plane. As a result, the projected intra-line intensity is modulated, as can be seen in Figure 3d. Due to the threshold nature of ablation, only regions exceeding the material threshold are ablated, yielding linewidths smaller than predicted by geometrical demagnification. This is consistent with the experimentally observed reduction factors (*k* < 1), i.e., ablated features that are systematically narrower than the nominal demagnified mask.

As a first estimate, the diffraction-limited lower bound on the resolvable feature size is given by the Rayleigh criterion a=0.61λ/NA≈ 520 nm, for a wavelength λ = 515 nm and a numerical aperture NA = 0.6. A more detailed resolution analysis of the projected line pattern can be assessed via the coherent cut-off of the 4f relay. For the axial separation between the tube lens and objective $z_{i}=320$ mm and an objective aperture stop diameter *D* = 12 mm, the coherent cut-off spatial frequency is $\nu_{c}=D/\left(2\lambda z_{i}\right)$. A line pattern approximated by a binary grating of period *p* has a fundamental spatial frequency $\nu_{g}=1/p$ that must satisfy $\nu_{g}<\nu_{c}$ (equivalently to $p>2\lambda z_{i}/D$) for the ±1 diffraction orders to pass through the pupil. This constraint prevents projecting a DMD line pattern with a linewidth of 13.68 µm (single-micromirror), corresponding to projected linewidths of 450 nm (M=1/30). Achieving single-micromirror line widths would require modifications to the experimental setup, in particular reducing the demagnification of the projected pattern or employing an objective with higher numerical aperture.

Further reduction of effective feature size can be achieved by reducing the fluence to levels just above the ablation threshold. At these fluences, the photoreaction and material removal are confined even more to the peak-intensity regions of the projected pattern, potentially yielding subdiffraction feature sizes [38]. Shorter illumination wavelengths (e.g., in the near-UV range) or projection optics with higher numerical aperture, together with an appropriate demagnification, provide a direct approach to improving resolution.

### 4.2. Scaling Potential

For the demonstrated concept enabling high-throughput and high-precision laser processing, a wide scan field, a large projected mask, adequate pulse energy, and high pattern rates are decisive. Together, these factors determine how effectively the architecture can be exploited.

The effective throughput in the scanning-based dynamic mask projection approach scales with the product of the projected mask area and the pattern rate (Equation (6)), whereby the pattern rate is set by the minimum of the DMD switching frequency, the laser repetition rate and the pattern-positioning rate. At the high DMD pattern and laser repetition rates of interest, repositioning is typically rate-limiting and is constrained by the dynamics of the positioning hardware. Throughput can therefore be increased by increasing the effective pattern-positioning speed, whereby galvanometer scanners provide higher accelerations and maximum velocities than linear translation stages.

Because throughput also scales with the projected mask area, maximizing the usable mask size directly increases processing speed. The maximum projectable mask area $A_{proj,max}$ is set by the maximum pulse energy and the required ablation fluence at the sample and can be estimated as:

(7) $$A_{proj,max}=\frac{E_{pulse,max}}{F}\cdot \eta_{opt},$$

where $E_{pulse,max}$ is the maximum pulse energy, $\eta_{opt}$ the overall optical efficiency and *F* the required processing fluence. However, the maximum pulse energy is limited not only by the laser source, but also by the requirement that the fluence on the DMD remains below its damage threshold, which strongly depends on the laser wavelength and pulse duration. In addition, the physical mask or DMD size sets a strict upper limit on the projected mask area for a given magnification:

(8) $$A_{proj,max}=A_{mask}\cdot M^{2},$$

where $A_{mask}$ denotes the DMD’s active area and *M* the system magnification factor. In the present optical configuration, the maximum projectable mask area is constrained by the available pulse energy (see Equation (7)), such that the full active area of the DMD cannot be exploited.

These considerations impose a clear constraint on the optical system: the scan field must exceed the projected mask area. Over extended scan fields, field-dependent loss of resolution can be mitigated by plan-corrected microscope objectives designed for wide fields of view, together with aberration-corrected tube and relay optics. Also, scan-angle-dependent vignetting should be minimized by relaying the scan mirror pivot plane to the objective entrance pupil and by limiting the objective–tube lens separation to prevent beam walk-off and the resulting pupil clipping, which would otherwise reduce the effective NA and thus the achievable resolution at large scan angles. In practice, the maximum usable projected field can also be constrained by field tilt. In the present setup, the DMD is illuminated at an oblique angle optimized for diffraction efficiency, which in combination with the projection system’s optical axis introduces a slight image tilt and axial focus variation at the workpiece. Owing to the strong demagnification and small projected mask area, this remains within the objective’s depth of focus. Even within the nominal depth of focus, the image-plane tilt introduces a systematic, monotonic defocus gradient across the projected field perpendicular to the tilt axis, thereby altering the local intensity distribution, consistent with the observed line-width variation along one axis in Figure 3c,e. For reducing this field-dependent defocus and enabling substantially larger projected fields, however, a projection axis perpendicular to the DMD would become necessary to maintain focus across the entire field.

To translate these results into an industrially viable system and to further optimize the proposed concept, several engineering aspects should be considered. In particular, deterministic synchronization of the laser pulses with the DMD patterns, the galvanometer scanner, and a planar two-axis translation stage enables dynamic and large-area processing. Within this synchronization scheme, a fixed delay implemented in the laser trigger path can compensate for deterministic latencies such as the micromirror switching time, thereby ensuring that each pulse is emitted within the DMD’s valid pattern display interval, defined as the time after micromirror settling and before the subsequent pattern transition. Timing constraints become increasingly critical at pattern rates substantially above those employed in this study, where the valid pattern display interval narrows as the pattern update period approaches the DMD switching overhead, or when timing jitter becomes comparable to the remaining timing margin. A second relevant engineering aspect concerns spatial placement (pattern stitching precision), which is influenced by both systematic and stochastic positioning effects. Deterministic field distortion of the galvanometer scan can, in principle, be reduced by scanner calibration and software-based pre-distortion. In contrast, the nearest-neighbor stitching-error statistics reported here quantify the residual local placement variability after removing systematic effects and thus indicate the practical stitching limit for applications requiring very high alignment precision. Additional throughput gains may be achieved using a hybrid scanning strategy, where a linear translation stage executes long strokes along one axis near its maximum travel while a galvanometer provides rapid orthogonal deflections of the projected pattern. This trajectory enlarges the addressable processing area and exploits the high deflection speed of the galvanometer scanner. Moreover, high-power ultrafast laser sources can enable a larger projected mask area, thereby further increasing parallelization and throughput. Beam shaping to achieve uniform DMD illumination further maximizes optical efficiency and suppresses intensity non-uniformities across the projected mask. Finally, focus stability across the entire sample must be ensured, and defocus must be compensated by axial adjustment if the depth of focus is exceeded.

### 4.3. Applications

The demonstrated method, dynamic mask projection combined with galvanometric scanning for ultrafast laser processing, is broadly applicable to processes requiring high-throughput, sub-micrometer, and selective material modification. Beyond generic micro- and nanostructuring, one particularly promising direction is long-term, high-density two-dimensional data storage in ceramic thin films where such a scanner-based system could be used to improve already demonstrated approaches [39,40]. Thereby, information is encoded by selective single-pulse ablation of projected binary patterns. Readout is achieved by bright-field microscopy due to the strong contrast between ablated and unmodified regions. Ceramic thin films, such as 10 nm TaN, are compelling candidates for durable, high-density storage media due to their comparatively low ablation thresholds, high optical contrast, and mechanical robustness [41], which supports long-term environmental stability. The achievable writing data rate scales with the digital dynamic mask’s array size (number of pixels) and pattern rate. Integrating a galvanometer scanner with high-NA, wide-field projection optics increases repositioning speed, enabling throughput to be maximized and approach the theoretical limit, described by the product of mask array size and the DMD’s switching frequency. Another rapidly emerging application domain is the fabrication of sub-micrometer structure arrays for metasurfaces, which enable advanced manipulation of electromagnetic waves across a broad range of optical and photonic applications [42,43,44,45,46,47]. Ultrafast laser ablation combined with SLM-based pattern projection has been demonstrated as a viable approach for fabricating such structures [27,28]. However, scalable methods for freely designed, large-area patterns that simultaneously achieve nanoscale feature sizes and high processing speeds remain limited. A similar need for high-throughput strategies is becoming increasingly critical in ultrafast laser additive manufacturing. As the field matures, industrial adoption and integration into commercial platforms will be largely governed by throughput [17]. These examples emphasize the need for scalable, high-throughput patterning in ultrafast laser processing; the scanning-based dynamic mask projection concept presented here addresses this gap.

Beyond the parameter range investigated here, the method can be extended to longer pulse durations, alternative laser wavelengths, and higher pulse energies, provided that the DMD and the optical components are operated below laser-induced damage thresholds and within thermal limits. Although demonstrated here on 10 nm TaN, the DMD-based mask projection and scanning concept is not inherently limited to this thickness or material system. For thicker films and/or materials with higher ablation thresholds, the processing window can be shifted towards higher peak fluences by increasing the pulse energy and/or by increasing the optical demagnification of the projected DMD pattern. Stronger optical demagnification reduces the projected pattern area, thereby increasing the fluence at the workpiece. In practice, this can be combined with illuminating a larger DMD area and applying micromirror binning to distribute the incident energy over a larger region on the DMD, thereby maintaining moderate fluence levels on the modulator while achieving higher fluence at the workpiece. In addition to this scalability in achievable fluence and material applicability, the scanning-based approach also offers practical system-level advantages. When the available scan field fully covers the target processing area, the workpiece can remain stationary and translational stages can be omitted. This is particularly beneficial for heavy or fragile substrates, as eliminating stage dynamics reduces mechanical risk and vibration coupling while simplifying system integration.

## 5. Conclusions

Scanning-based dynamic mask projection enables high-throughput sub-micrometer ablation of thin films by combining a DMD-defined binary amplitude mask with galvanometric scanning through a high-NA objective. Using 230 fs pulses at 515 nm, selective single-pulse ablation of 10 nm TaN on glass was achieved with minimum linewidths of 770 nm, consistent with Fourier-optical simulations that account for diffraction and the objective’s finite numerical aperture. By addressing the throughput–resolution trade-off inherent to ultrafast laser writing, the proposed architecture achieves an areal throughput of $3.1\;{\mathrm{mm}}^{2}/s$ for sub-micron features, representing a 20× increase over single-spot scanning under comparable conditions. Scan field characterization shows that the usable processing area is ultimately constrained by off-axis aberrations and system apertures; an equivalent circular scan field diameter of 550 µm was achieved. The results further indicate that scanning-based dynamic mask projection offers benefits beyond spatial parallelization. At typical DMD switching frequencies and laser repetition rates, lateral pattern repositioning becomes rate-limiting, thus constraining throughput. Galvanometric scanning, providing accelerations and peak velocities that substantially exceed those of linear translation stages, mitigates this limitation within the objective’s field of view. The scalability analysis highlights key engineering requirements for transferring the concept toward larger-area, industrially relevant implementations: (i) wide-field, flat-field-corrected, high-NA relay/projection optics to provide a scan field larger than the projected mask area while preserving resolution across the field, and (ii) deterministic synchronization of laser, DMD, galvanometer, and a 2D translation stage to enable hybrid trajectories that combine long-stroke travel with fast orthogonal deflection. With these measures, scanning-based dynamic mask projection provides a practical route to high-speed, reconfigurable micro- and nanoprocessing, supporting application domains including metasurface manufacturing, high-throughput selective thin film processing, and long-term optical data storage.

## Figures and Tables

**Figure A1:**
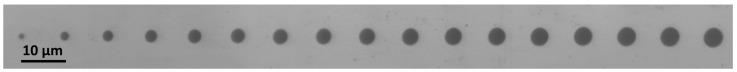
High-resolution optical microscopy image of single-shot ablation spots on 10 nm TaN coated glass for increasing pulse energies (left to right), resulting in increasing spot diameters used for determining the single-pulse ablation threshold at 515 nm with a 230 fs pulse duration.

**Figure A2:**
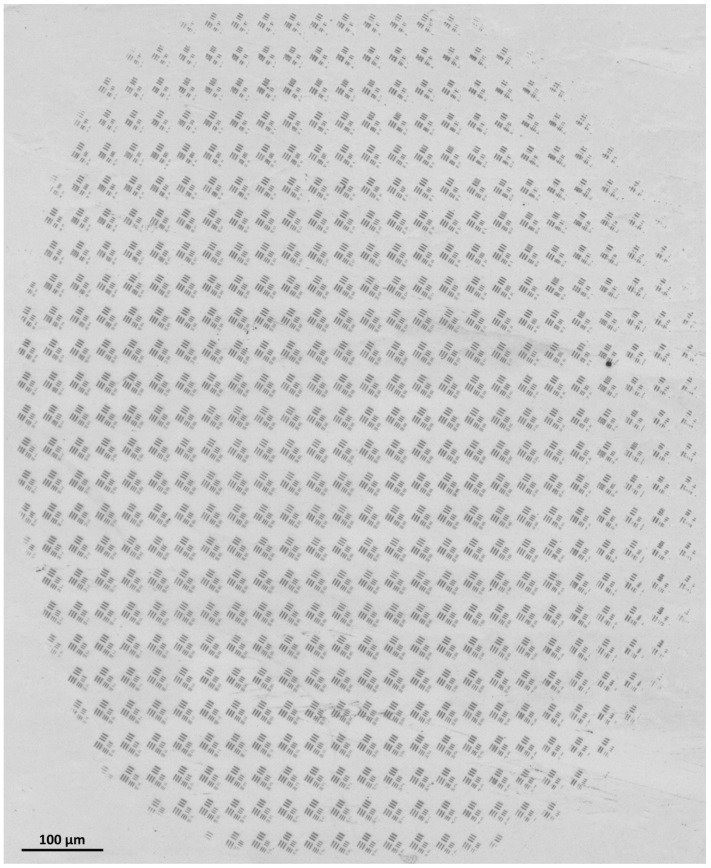
Line patterns with linewidths of (0.77±0.05), (1.26±0.08), and (1.61±0.17) µm are scanned across the maximum scan field contrained by the size of the sytem’s apertures and ablated with single pulses on a 10 nm TaN thin film sample. Resolution degrades with increasing distance from the field center.

## Data Availability

The original contributions presented in this study are included in the article. Further inquiries can be directed to the corresponding author.

## Appendix A

### Appendix A.1. Single-Shot Ablation Threshold by Liu’s Method

Single-shot ablation threshold characterization was performed for 230 fs, 515 nm pulses following Liu’s [31] linear regression method. The sample’s surface was irradiated with single pulses at 20 sites across the sample and the pulse energies were varied gradually across discrete levels, not all exceeding modification threshold. Ablated spots on the sample surface were examined using optical microscopy with a high-resolution objective, see Figure A1. The measured diameters of the ablated spots and corresponding pulse energies are given in Table A1.

**Table A1:** Measurements for single-shot ablation threshold determination of 10 nm TaN coated glass substrates at 515 nm with 230 fs pulses; ablated spot diameters and corresponding single-pulse energies.

Ablated Spot Diameter *D* (µm)	Pulse Energy *E_pulse_* (nJ)
1.51	16.0
2.35	19.8
2.75	23.7
3.06	27.4
3.39	31.2
3.50	34.8
3.63	38.5
3.81	42.2
3.98	45.8
4.12	49.5
4.21	53.2
4.31	56.8
4.45	60.3
4.48	63.8
4.58	67.3
4.68	70.6
4.76	73.8

### Appendix A.2. Maximum Scan Field

The extent of the scan field is primarily constrained by the clear apertures of the system mirrors and relay optics, as well as by the objective’s field stop. The aperturization introduced by the final 90° deflection at a circular folding mirror is responsible for the elliptical shape of the processing area, as shown in Figure A2.
